# The Invisible threshold of childbearing: an investigation of social trust and fertility intentions

**DOI:** 10.3389/fpsyg.2026.1833532

**Published:** 2026-08-05

**Authors:** Huanjun Li

**Affiliations:** School of Ethnology and Sociology, Minzu University of China, Beijing, China

**Keywords:** expert-system trust, fertility intentions, institutional trust, interpersonal trust, subjective wellbeing

## Abstract

**Introduction:**

Against the backdrop of persistently low fertility in China, understanding the psychosocial factors associated with fertility intentions has become increasingly important for both demographic research and policy development.

**Methods:**

Drawing on nationally representative data from the 2023 Chinese General Social Survey (CGSS), this study examines the associations between multidimensional social trust and fertility intentions and further explores the mediating role of subjective well-being. Building on trust theory, social trust is conceptualized as a multidimensional construct comprising institutional trust, interpersonal trust, and expert-system trust, with interpersonal trust further distinguished into particularized trust and generalized trust. Ordered logistic regression and structural equation modeling are employed to examine these relationships.

**Results:**

The results indicate that institutional trust and expert-system trust are positively associated with fertility intentions, and these associations are partially mediated by subjective well-being. Particularized trust shows no significant direct association with fertility intentions but is indirectly associated with fertility intentions through subjective well-being. By contrast, generalized trust is not significantly associated with fertility intentions. These findings suggest that different forms of social trust are associated with fertility intentions through distinct psychological and social pathways.

**Discussion:**

By distinguishing expert-system trust from institutional trust and identifying subjective well-being as an important psychological mechanism, this study extends the multidimensional analytical framework of social trust and contributes to a better understanding of fertility intentions in contemporary China. The findings also provide evidence that may inform the design of policies aimed at creating a more supportive institutional and social environment for family formation.

## 1 Introduction

Over the past decade, China has entered a stage of persistently low fertility, with the total fertility rates remaining well below the replacement level despite successive policy adjustments (Jiang et al., 2016). Following the transition from the one-child policy to the universal two-child policy in 2016 and subsequently to the three-child policy in 2021, the Chinese government has increasingly introduced a series of pronatalist measures, including institutional reforms, expanded childcare services, and family-support policies. However, empirical evidence suggests that these reforms have not been accompanied by a sustained recovery in fertility levels (Yang et al., 2023; Zhou and Guo, 2020). This divergence between policy intention and demographic outcomes suggests that fertility decline cannot be explained solely in terms of policy restrictions or economic incentives (Zhang et al., 2023). In low-fertility societies, childbearing increasingly reflects a deliberate and future-oriented decision made under conditions of economic, institutional, and social uncertainty. Consequently, understanding fertility intentions requires moving beyond policy design to examine how individuals perceive future risks, social support, and institutional reliability when making reproductive decisions.

Fertility intentions constitute an important antecedent of childbearing behavior, reflecting individuals’ expectations and preferences regarding future parenthood. A substantial body of demographic research has shown that fertility intentions are strongly associated with subsequent childbearing behavior (Schoen et al., 1999; Lau et al., 2018), particularly in low-fertility societies where childbearing is increasingly driven by deliberate choice rather than normative obligation. In the Chinese context, rapid socioeconomic transformation, including rising educational attainment, urbanization, and increasing individualization, has fundamentally reshaped life-course expectations and family formation patterns. Against this backdrop, fertility intentions provide a valuable analytical lens for understanding reproductive decision-making. Rather than focusing solely on policy interventions or economic constraints, an examination of fertility intentions makes it possible to explore how individuals evaluate the desirability, uncertainty, and perceived costs of childbearing.

Existing research has identified a wide range of factors associated with fertility intentions. At the individual and family level, socioeconomic characteristics, economic resources, and family relationships have received the greatest attention. Previous studies have consistently shown that gender, educational attainment, employment conditions, and financial pressures are closely associated with fertility intentions (Fan and Maitra, 2010; Testa and Stephany, 2017; Kuhnt and Trappe, 2016; Yu and Kuo, 2017; Fiori et al., 2013). Family-level characteristics, including marital satisfaction, family support, intimate relationships, parental attitudes toward childbearing, the number of siblings, and family size, have also been found to be related to reproductive preferences (Berninger et al., 2011; Hanappi et al., 2017; Kotte and Ludwig, 2011). From a sociocultural perspective, the rise of individualistic values has transformed childbearing from a life necessity into a lifestyle choice (Testa and Rampazzo, 2017). Awareness of gender equality, religious beliefs, and family policy arrangements have likewise been linked to fertility intentions (Rosina and Testa, 2009; Buber et al., 2013).

Despite these important contributions, much of the existing literature conceptualizes institutional and social environments as exogenous background conditions while paying comparatively less attention to how individuals subjectively perceive these environments. In particular, limited attention has been paid to whether trust in institutions, professionals, and social relationships shapes individuals’ perceptions of uncertainty and future stability, both of which are likely to be closely associated with fertility preference. This gap is particularly salient in contemporary China, where rapid socioeconomic transformation has simultaneously reconfigured institutional arrangements, professional service systems, and interpersonal networks.

Social trust provides an important analytical perspective for addressing this gap. As a key component of social capital (Bain and Hicks, 1998), social trust reflects individuals’ expectations regarding cooperation, reciprocity, and institutional reliability (Knack, 2002). Several studies report positive associations between social trust and fertility-related outcomes. Cross-national studies have shown that societies characterized by higher levels of social trust generally exhibit relatively higher fertility rates (Yamamura and Antonio, 2011). Similar evidence has been reported in South Korea, where social trust is positively associated with young adults’ fertility intentions (Kwag et al., 2024). In China, Ning et al. (2022) revealed that social trust positively mediates the relationship between media use and fertility intentions among women of childbearing age. Collectively, these studies suggest that social trust constitutes an important social resource that may shape fertility preference by reducing uncertainty and strengthening expectations about future societal conditions.

However, the existing fertility literature has largely treated trust as either a unidimensional concept or a broad contextual factor, leaving limited room to examine whether distinct trust configurations exhibit heterogeneous associations with fertility intentions. Trust theory suggests that trust is directed toward different social objects rather than representing a single generalized orientation. Luhmann (1968) conceptualizes trust as a mechanism for reducing social complexity and distinguishes between personal trust and system trust, with the latter referring to trust in impersonal social and institutional systems that sustain social order. Building on this perspective, Giddens (1990) further develops the concept of expert systems, arguing that modern societies increasingly rely on trust in professional knowledge and technical expertise to cope with uncertainty. Although institutional trust and expert-system trust both represent forms of system trust, they are directed toward different targets. Recent scholarship has noted that the conceptual relationship between these two forms of trust warrants further clarification (Valgarğsson et al., 2025), particularly in contexts where professional systems are closely intertwined with state governance. This issue is especially salient in China, where professionals such as doctors, teachers, judges, and bank employees are often embedded within highly institutionalized and state-regulated organizations. Consequently, trust in these professionals may partially reflect broader trust in public institutions. Nevertheless, such institutional overlap does not imply conceptual equivalence. Institutional trust concerns trust in the legitimacy and effectiveness of formal institutions, whereas expert-system trust concerns confidence in the competence and reliability of professionals who provide specialized knowledge and services (Giddens, 1990). Although institutional trust and expert-system trust are expected to be positively correlated in China, they represent distinct dimensions through which individuals manage uncertainty. Therefore, this study conceptualizes expert-system trust as an institutionally embedded but analytically distinct dimension of social trust, whose empirical distinctiveness is examined in the subsequent analysis. Together, institutional trust, interpersonal trust, and expert-system trust constitute a multidimensional framework for examining how different types of trust are associated with fertility intentions.

### 1.1 Institutional trust

Consistent with the conceptual distinction discussed above, institutional trust refers to individuals’ confidence in the legitimacy, effectiveness, and reliability of formal state institutions engaged in governance, law enforcement, and public service provision (Kaasa and Andriani, 2022). Yang and Tang (2010) conceptualize institutional trust as a multidimensional construct comprising administrative trust, legal trust, and societal trust, highlighting its heterogeneous character across institutional domains. In the context of demographic transition, institutional trust can be understood as an indicator of individuals’ confidence in public institutions to provide stable policy environments and reliable welfare support. Individuals with higher levels of institutional trust may therefore feel more secure about childcare support, healthcare accessibility, and pension security, thereby reducing perceived structural uncertainty surrounding childbearing.

However, existing evidence on the relationship between institutional trust and fertility intentions remains mixed. Several studies suggest that institutional trust facilitates fertility intentions by increasing confidence in public services and family-support policies. For example, Gortfelder et al. (2024) differentiate between interpersonal and institutional trust and argue that institutional trust increases individuals’ willingness to rely on external childcare services and medical resources (Aassve et al., 2016). When couples depend on such services, the perceived opportunity costs of childbearing are reduced, and fertility-related concerns may decrease, potentially enhancing fertility intentions. In contrast, Zheng et al. (2024) report a negative association between individuals’ trust in formal institutions (e.g., local governments) and fertility intentions in China, and find that greater trust in public institutions may reduce individuals’ reliance on children as a source of old-age security (Yeh, 2022). Taken together, these inconsistent findings suggest that the relationship between institutional trust and fertility intentions is contingent upon broader institutional and policy contexts.

China provides a particularly important setting for examining this relationship. Higher levels of institutional trust exist alongside the long-term influence of the one-child policy, which has contributed to relatively low fertility expectations. Since the introduction of the universal two-child policy in 2016 and the three-child policy in 2021, the Chinese government has increasingly shifted its stance from fertility restriction to fertility promotion. This shift is reflected in a range of family-support initiatives. Under this evolving policy environment, institutional trust may increase expectations regarding the implementation and credibility of such supportive policies, and thus shape fertility intentions in different ways. Accordingly, higher institutional trust is expected to be associated with stronger fertility intentions in contemporary China. Thus, we proposed:

*H1*: Institutional trust is positively associated with fertility intentions in China.

### 1.2 Interpersonal trust

Interpersonal trust captures trust in other individuals and is embedded in ongoing social relationships and everyday social interactions. Following the distinction commonly adopted in the social capital literature, this dimension can be further divided into particularized trust—trust that is confined to close-knit networks such as family members, friends, and colleagues—and generalized trust, which extends to unfamiliar others and society more broadly. Although both dimensions reflect confidence in other individuals, they operate through different mechanisms and have distinct implications for fertility decision-making.

Particularized trust reflects expectations of emotional and instrumental support within close social networks. Such support can reduce both the practical and psychological costs of child-rearing by increasing confidence that childcare responsibilities and family obligations can be shared. Previous research has shown that family environments and close social relationships play an important role in shaping fertility preferences. For instance, parental attitudes toward fertility, the number of siblings, and other family-related factors tend to be transmitted across generations, thereby influencing individuals’ fertility decisions (Zheng et al., 2024). Moreover, fertility intentions and behaviors are “contagious” within peer groups such as friends and colleagues (Hensvik and Nilsson, 2010). Earlier research has found that more supportive network relationships are positively related to fertility intentions (Philipov et al., 2006). When individuals place higher trust in close family members and friends, they are more likely to anticipate greater social and emotional support, which in turn strengthens their fertility intentions.

Generalized trust, by contrast, reflects confidence in unfamiliar others and the broader social environment. Rather than being anchored in close personal relationships, generalized trust captures perceptions of social cohesion, reciprocity, and collective reliability. Higher levels of generalized trust may reduce perceived social risks and enhance confidence in the wider social environment, thereby creating a more favorable context for family planning. Some studies have also found that generalized trust is positively associated with fertility attitudes (Alijanzadeh et al., 2023), whereas lower levels of generalized trust may undermine perceived personal security and reduce willingness to have children (Ghaferi et al., 2020). Taken together, these findings suggest that both dimensions of interpersonal trust are expected to facilitate fertility intentions, albeit through different social mechanisms. Accordingly, this study proposed these two hypotheses:

*H2a*: Particularized trust is positively associated with fertility intentions in China.

*H2b*: Generalized trust is positively associated with fertility intentions in China.

### 1.3 Expert-system trust

Beyond institutional and interpersonal domains, contemporary differentiated societies rely heavily on abstract systems grounded in professional knowledge and technical expertise. Drawing on Giddens’ notion of expert systems (Giddens, 2002), this study introduces expert-system trust to capture confidence in professionals and specialized systems that provide essential public services, such as medical professionals, educators, and financial experts. Unlike institutional trust, which concerns trust in the legitimacy and effectiveness of formal institutions, expert-system trust reflects perceptions of competence, reliability, and professionalism among service providers. Although professionals in China are largely embedded within state-regulated institutions, an analytically distinct dimension of trust can still be distinguished.

In highly differentiated societies, reproductive decisions are increasingly contingent upon trust in expert systems, as childbearing entails long-term commitments that depend heavily on reliable access to healthcare, education, childcare, and financial planning. Uncertainty in these domains can significantly constrain decisions about union formation and childbearing (Kohler et al., 2002; Comolli, 2017). Individuals who trust expert systems are therefore more likely to expect that essential services will function effectively when needed, thereby increasing perceived capacity and sense of security regarding child-rearing. Accordingly, higher levels of expert-system trust are expected to be positively associated with fertility intentions. This leads to the following hypothesis:

*H3*: Expert-system trust is positively associated with fertility intentions in China.

### 1.4 Subjective wellbeing

In exploring the relationship between social trust and fertility intentions, this study further focuses on subjective wellbeing as a key mediating factor. Subjective wellbeing refers to individuals’ overall evaluation of their quality of life and psychological security and has been widely recognized as an important determinant of fertility-related decision-making. Aassve et al. (2015) demonstrate that subjective wellbeing plays a crucial role in shaping fertility decisions. Higher levels of happiness increase both the willingness and the likelihood of having children, which in turn contribute to higher fertility rates at the societal level. Hobcraft and Kiernan (1995) propose that prospective parents face five basic needs when deciding to have children, among which subjective wellbeing, as a form of psychological security, naturally encourages childbearing decisions. Consistent with this view, wellbeing not only increases individuals’ willingness to have children but also encourages them to have additional children (Perelli-Harris, 2006). Billari (2009) similarly argues that the quest for happiness is a common factor guiding fertility in contemporary societies, with childbearing both reflecting and contributing to individuals’ perceived life satisfaction. Existing research has explored this relationship from multiple perspectives. On the one hand, individuals with higher levels of happiness tend to exhibit stronger positive attitudes toward future life. Affective forecasting theory suggests that individuals make decisions based on affective forecasts, and those who feel overall wellbeing are more likely to prepare for the responsibilities of parenthood (Mencarini et al., 2018). On the other hand, well-being is often associated with higher-quality marital relationships and more stable family environments, which provide an important foundation for successful child-rearing (Tan and Anwar, 2025). Consequently, subjective wellbeing provides an important psychological foundation for fertility intentions.

Social trust is also consistently associated with higher levels of subjective wellbeing. Research across various countries consistently demonstrates the positive impact of social trust on individual wellbeing (Lu et al., 2020; Kuroki, 2011; Zhou et al., 2021; Tokuda et al., 2010). Helliwell and Putnam (2004), utilizing data from the World Values Survey, the US Benchmark Survey, and a comparable Canadian survey, show that trust, as a form of social capital, is independently and robustly correlated with happiness and life satisfaction. In a subsequent study, Helliwell et al. (2017) compare data from three large international surveys and reaffirm the centrality of trust to wellbeing. This effect is observed both at the national and individual levels, with individuals residing in higher-trust environments reporting significantly higher subjective wellbeing. Additionally, when individuals exhibit greater trust in colleagues, neighbors, police, and strangers, they tend to report higher levels of subjective wellbeing (Helliwell and Wang, 2011). Helliwell et al. (2012) further argue that the stagnation in life satisfaction in the United States and the United Kingdom, in contrast to the increase observed in many European countries, is largely attributed to the decline in the quality of interpersonal relationships, including diminished trust. Similarly, data from China also reveal a significant positive effect of social trust on subjective wellbeing, with results corroborated even when distinguishing between particularized trust and general trust (Gao and Zhao, 2022; Li and He, 2022).

Recent studies further suggest that different forms of trust may contribute to wellbeing through distinct pathways. Helliwell et al. (2017) distinguish different forms of trust and find that trust in police plays the most significant role in promoting wellbeing. Furthermore, they identify significant additional benefits from trust in three key aspects of the institutional environment: the legal system, parliament, and politicians. Cross-national evidence from 97 countries reveals that trust in government is positively associated with subjective wellbeing both directly and indirectly through improvements in broader socioeconomic conditions (Suriyanrattakorn and Chang, 2021). Based on these findings, and in alignment with the theoretical arguments, we proposed four specific hypotheses as follows:

*H4*: Subjective wellbeing mediates the relationship between institutional trust and fertility intentions.

*H5a*: Subjective wellbeing mediates the relationship between particularized trust and fertility intentions.

*H5b:* Subjective wellbeing mediates the relationship between generalized trust and fertility intentions.

*H6:* Subjective wellbeing mediates the relationship between expert-system trust and fertility intentions.

## 2 Data and research strategy

### 2.1 Data and methods

The data used in this study are drawn from the 2023 Chinese General Social Survey (CGSS), conducted by the China Survey and Data Center at Renmin University of China. Since 2003, the CGSS has surveyed more than 10,000 respondents across mainland China, covering social, community, family, and individual dimensions. The survey employs a nationally and provincially representative sampling design and is widely recognized as one of the most authoritative social surveys in China. The CGSS2023 dataset initially included 11,326 respondents. After restricting the sample to respondents with complete information on social trust and fertility intentions and excluding observations with missing or invalid values, the final analytical sample consisted of 5,455 respondents. The respondents in our sample ranged in age from 18 to 80, covering individuals across different stages of the life course. Among these respondents, 2,418 were male (44.33%), and 3,037 were female (55.67%). The married population was 4,039 (74.0%), and the unmarried population was 1,416 (25.96%). The number of urban was 2,284 (42.03%), and the number of rural was 3,150 (57.97%).

To examine whether the exclusion of respondents with missing information introduced potential selection bias, we compared the demographic characteristics of the full CGSS2023 sample (*N* = 11,326) with the final analytical sample (*N* = 5,455), using standardized mean differences (SMDs) and intergroup significance tests. The results showed that no significant systematic imbalance was observed in the demographic characteristics between our sample and the full sample, indicating that sample restriction did not substantially alter the composition of respondents or generate serious selection bias. It should be mentioned that since this survey was conducted during the COVID-19 pandemic, data were collected through both in-person visits and telephone interviews. However, questions regarding social trust were not included in the telephone interviews, which involved 5,743 respondents. Therefore, the sample we used does not affect the representativeness of the analysis sample.

We employed ordinal logistic regression and structural equation modeling to examine the associations between different dimensions of social trust and fertility intentions, as well as the mediating role of subjective wellbeing. First, ordinal logistic regression models were estimated using Stata MP 17 to analyze the basic relationship between different dimensions of social trust and fertility intentions. Second, structural equation modeling was employed to explore whether subjective wellbeing mediated the association between social trust and fertility intentions. Additionally, missing data were handled using multiple imputation to reduce potential bias arising from missing responses. Observations with extensive missing values were excluded prior to imputation.

### 2.2 Dependent variable

Fertility intention is the dependent variable in this study, which is measured as respondents’ ideal number of children. Specifically, respondents were asked: “If there were no policy restrictions, how many children would you ideally like to have?” Respondents provided an open-ended numeric answer indicating their desired number of children. In order to facilitate the subsequent statistical analysis and to align with the requirements of ordinal logistic regression, the missing and extreme values were excluded, and fertility intention was recoded into four categories: 0 = no desire for children, 1 = desire for one child, 2 = desire for two children, and 3 = desire for three or more children.

It should be noted that this measure captures individuals’ ideal fertility preferences rather than immediate childbearing plan. Although respondents beyond reproductive ages may have completed their childbearing trajectories, their responses remain informative because they reflect relatively stable fertility norms, family values, and retrospective evaluations of reproductive preference. Previous demographic studies have similarly employed ideal family size measures among broad adult populations to examine fertility preferences at the societal level (Hin et al., 2011). Therefore, the present study interprets fertility intention as a measure of desired fertility preferences and focuses on its association with social trust rather than directly predicting subsequent fertility behavior.

### 2.3 Independent variables

The independent variable in this study is social trust, measured by the CGSS2023 survey question: “To what extent do you trust the following people?” The question includes 17 trust targets: relatives, friends, neighbors, colleagues, strangers, doctors, bank clerks, corporate executives, journalists, leaders of NGOs (non-governmental organizations), teachers, local officials, central officials, police officers, military officers, judges, and online friends. Responses were coded as 1 = completely distrust, 2 = somewhat distrust, 3 = somewhat trust, and 4 = completely trust.

The KMO measure of sampling adequacy was 0.894, and Bartlett’s test of sphericity yielded a chi-square value of 37,365.698, with 136 degrees of freedom (*p* < 0.001), indicating that the data were suitable for factor analysis. Based on exploratory factor analysis, four main factors were extracted and labeled as institutional trust, particularized trust, generalized trust, and expert-system trust (Table 1). These factors jointly accounted for 61.84% of the total variance. The minimum factor loading was 0.476, and all other loadings exceeded 0.55, suggesting a generally adequate level of indicator contribution to the latent constructs. To further assess the robustness of the proposed factor structure, confirmatory factor analysis was conducted. The model fit indices indicate an acceptable fit. The confirmatory fit index (CFI) value was 0.905, the Tucker-Lewis index (TLI) value was 0.895, the root mean squared error of approximation (RMSEA) value was 0.045, and the standardized root mean squared residual (SRMR) was 0.040. These values fall within commonly accepted thresholds for model adequacy.

**TABLE 1:** Items and component matrices in social trust.

Items	Means	SD	Rotated factor component matrix			
			Institutional trust	Particularized trust	Generalized trust	Expert-system trust
Teachers	3.199	0.647	0.550			
Local officials	2.913	0.760	0.694			
Central officials	3.186	0.724	0.763			
Police officers	3.167	0.696	0.866			
Military officers	3.234	0.668	0.842			
Judges	3.087	0.728	0.823			
Relatives	3.317	0.605		0.749		
Friends	3.150	0.606		0.833		
Neighbors	3.044	0.608		0.791		
Colleagues	2.859	0.644		0.627		
Strangers	1.654	0.654			0.785	
Online friends	1.537	0.670			0.779	
Doctors	3.072	0.697				0.476
Bank clerks	2.830	0.705				0.717
Corporate executives	2.467	0.742				0.753
Journalists	2.592	0.746				0.699
Leaders of NGOs	2.406	0.788				0.590
Variance			3.970	2.528	1.490	2.524
Cumulative			61.84%			
Method			Principal-component factors			

### 2.4 Mediating variable

The mediating variable in this study is subjective wellbeing, which refers to an individual’s overall perception and evaluation of their quality of life. The specific item used to measure subjective wellbeing in the survey is: “Overall, how happy do you feel with your life?” This item is widely used as a proxy for subjective wellbeing in survey research. The responses were categorized into five levels according to the Likert scale, ranging from “very unhappy” to “very happy”, corresponding to scores from 1 to 5.

### 2.5 Control variables

In this study, we controlled for a set of sociodemographic and contextual variables, including gender (male = 1; female = 0), age, ethnicity (Han = 1, non-Han = 0), political affiliation (Chinese Communist Party / CCP member = 1, non-CCP member = 0), marital status (married = 1, unmarried = 0), years of education, personal income, place of residence (urban = 1, rural = 0), employment status (employment = 1, unemployment = 0), and self-rated health.

Years of education were operationalized by converting respondents’ highest level of educational attainment into years based on standard assumptions in the Chinese education system: no formal education was coded as 0, traditional private school or literacy class as 3, primary school as 6, junior high school as 9, senior high school or vocational school as 12, junior college as 15, undergraduate degree as 16, and postgraduate degree or above as 19. Personal income was measured in logarithmic form, using self-reported total annual personal income from the previous year. Considering the relatively high level of social mobility in China, many residents with rural hukou work and live in urban areas, where their actual social interactions and living conditions are more closely tied to urban settings. As hukou status may not fully capture the living circumstances of individuals (Wang et al., 2025), and given substantial missing data on this variable in the dataset, place of residence was used as an alternative proxy. Self-rated health was included as a key control given its potential relevance to fertility behaviors, and was categorized into five levels, with higher values indicating better health. Other potential confounding factors, such as region (or province), were excluded from consideration due to data limitations.

## 3 Results

### 3.1 Descriptive statistical analysis

#### 3.1.1 Social trust and its generational change in china

**FIGURE 1:**
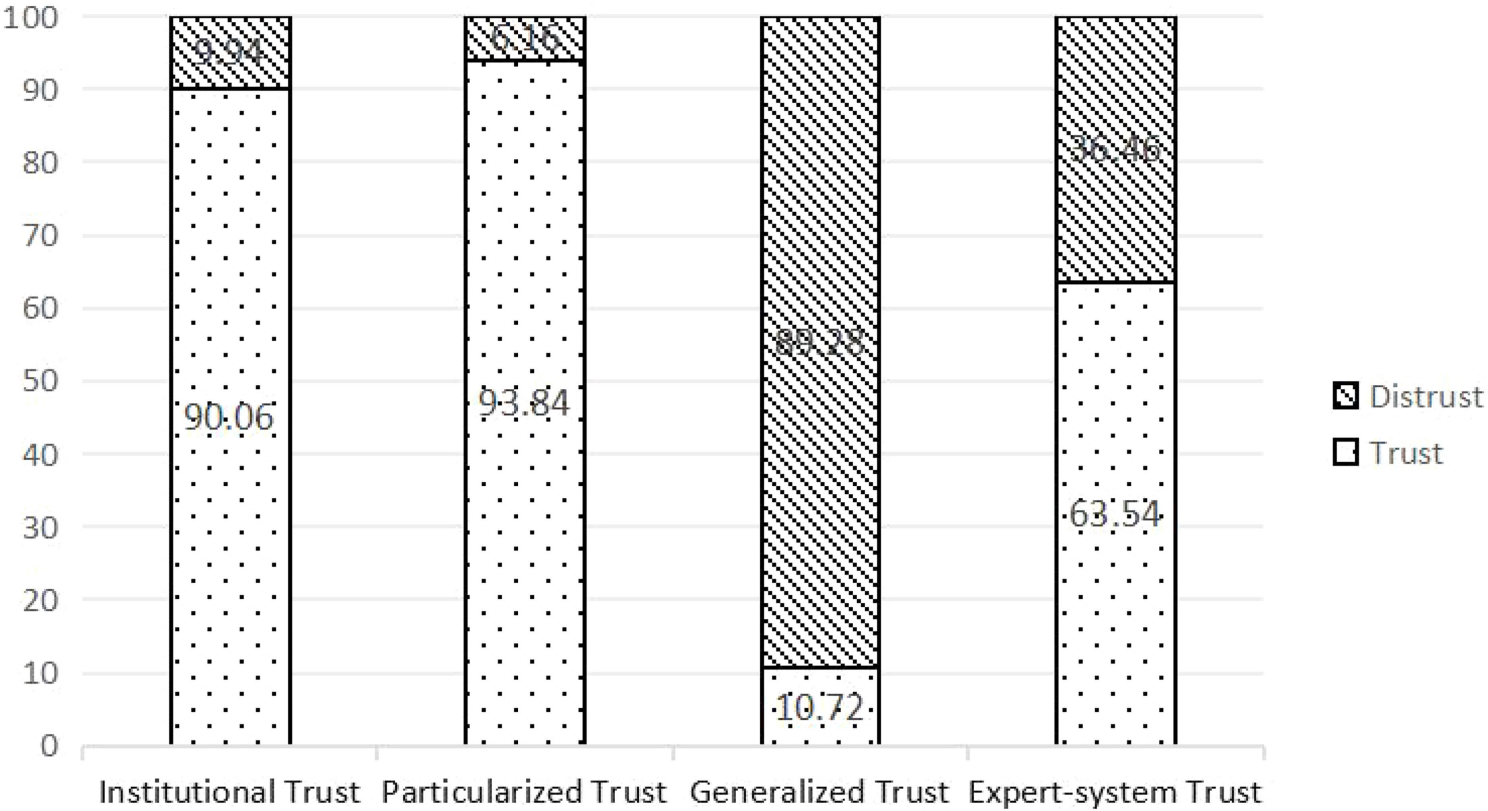
Categories and degrees of social trust.

Figure 1 presents the distribution of the four dimensions of social trust. Overall, respondents report the highest level of particularized trust, with 93.84% of individuals expressing trust in acquaintances. In Chinese social relations, family members and close friends constitute individuals’ closest social relationships, and thus naturally command the highest level of trust. Drawing on Fei Xiaotong’s conceptualization of Chinese social relations as a “differential mode of association” (water ripple model), social relationships are organized in concentric circles based on relational proximity, with family and friends located at the innermost level of the network. Within this relational framework, trust is expected to decline as social distance increases. Institutional trust is somewhat lower than particularized trust, but still remarkably high at 90.06%. This high level of institutional trust has been partly interpreted as reflecting long-standing cultural traditions emphasizing respect for authority and hierarchical order, as discussed in Confucian thought (Fung and Etienne, 2023). Expert-system trust is comparatively low, with 63.54% of respondents reporting trust. Generalized trust is the lowest, with only 10.72% expressing trust in strangers. Data in Table 1 further show that trust in online friends (mean = 1.537) is even lower than trust in strangers (mean = 1.654). With the expansion of social interactions through technological advances and the widespread use of internet platforms, individuals’ social networks have broadened. However, insufficient regulation of online platforms and the uncertainty surrounding online information have gradually eroded trust in virtual social interactions.

**FIGURE 2:**
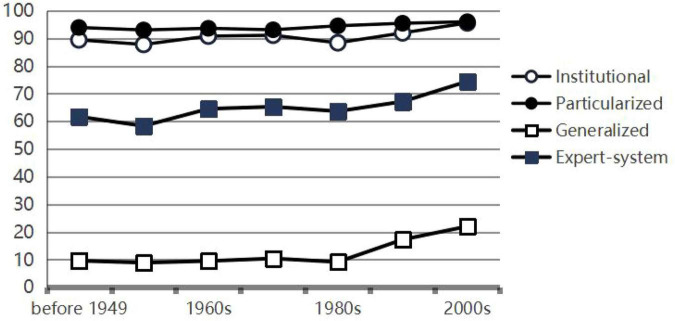
Cohort differences in social trust.

Figure 2 illustrates cohort differences in four types of social trust. Institutional trust and particularized trust remain consistently high and stable across birth cohorts. In contrast, expert-system trust and generalized trust—though differing in levels—exhibit broadly similar cohort patterns. For cohorts born before the 1980s, trust levels show strong homogeneity with little variation; however, for cohorts born in the 1980s and later, trust in both experts and individuals in general appears to increase to some extent. More than 70% of those born in the 2000s express trust in the expert system, while approximately 20% report generalized trust. These findings suggest that, with greater social openness and accelerated information flows, younger generations display higher levels of trust in diverse environments and groups. The evolution of social structures and cultural norms under globalization has fostered a tendency among younger generations toward open interaction and broader social trust networks, even though the foundation of such trust has yet to be fully consolidated.

#### 3.1.2 Fertility intentions and group differences in china

Table 2 presents the basic patterns of fertility intentions in China. Overall, the mean level of fertility intention in China is 1.942. More than half of the respondents report a preference for having two children, reflecting the enduring cultural preference for a “balanced” family structure in China, often symbolized by the ideal of having both a son and a daughter. More than one in five respondents prefer having three or more children, while only 6.4% report a preference for having no children. These patterns suggest that the ideal family size among Chinese adults remains largely centered around the two-child norm, although considerable variation exists across individuals.

**TABLE 2:** Descriptive statistical analysis of fertility intentions (*N* = 5,455).

Variable		Freq.	Percent(%)
Fertility intentions	0	349	6.40
	1	797	14.61
	2	3,131	57.40
	3	1,178	21.59
Variables		Fertility intentions Mean (SD)/Corr.	
Gender	Male	2.005 (0.016)	
	Female	1.892 (0.014)	
Age		0.332***	
Ethnicity	Han	1.929 (0.011)	
	Non-Han	2.067 (0.030)	
Political affiliation	CCP member	1.959 (0.030)	
	Non-CCP member	1.940 (0.011)	
Marital status	Married	2.025 (0.011)	
	Unmarried	1.706 (0.025)	
Years of education		−0.281***	
Personal income		−0.049***	
Place of residence	Urban	1.752 (0.017)	
	Rural	2.079 (0.013)	
Employment status	Employment	1.905 (0.015)	
	Unemployment	1.975 (0.015)	
Health		−0.108***	

***p < 0.001.

Fertility intentions tend to increase with age, while years of education are negatively correlated with fertility intentions, indicating that individuals with higher educational attainment tend to report weaker fertility intentions. This finding is consistent with prior research (Gobbi, 2012; Waren and Pals, 2013). With regard to income, Li (2023) argues that income is positively associated with fertility intentions, as higher income improves the ability to bear child-rearing costs. However, this study suggests an alternative view: fertility intentions are negatively associated with personal income. We posit that social development typically coincides with higher education, higher income, and lower fertility intentions, as improvements in living standards encourage individuals to prioritize personal development and quality of life, thereby reducing fertility intentions.

The results also reveal group-level differences. Males report stronger fertility intentions than females. Married individuals show substantially stronger fertility intentions than unmarried individuals, suggesting that in Chinese society, marriage and childbearing remain closely interconnected. Fertility intentions among urban residents are weaker than those among rural residents, reflecting the impact of urbanization on fertility attitudes and intentions. Fertility intentions among Han respondents are slightly weaker than those of ethnic minorities, while no significant difference is observed between CCP members and non-CCP members, or between employed and unemployed respondents. Interestingly, self-rated health is negatively associated with fertility intentions, a pattern that suggests further interpretation in light of potential heterogeneity in fertility planning and life-course considerations.

### 3.2 Correlation analysis

Table 3 presents the correlations among social trust, subjective wellbeing, and fertility intentions. Overall, the relationships between different types of social trust and fertility intentions vary considerably. Institutional trust, particularized trust, and expert-system trust are all positively and significantly correlated with fertility intentions, with institutional trust exhibiting the strongest correlation (*r* = 0.096), indicating that it may be more closely related to fertility intentions compared with the other trust dimensions in the bivariate framework. Expert-system trust shows the weakest correlation (*r* = 0.051), yet still demonstrates a positive association. In contrast, generalized trust is significantly negatively correlated with fertility intentions (*r* = −0.091), suggesting that higher levels of trust in strangers and online friends are associated with weaker fertility intentions. One possible explanation is that modernization has promoted individual autonomy, particularly among women, who increasingly emphasize personal development and self-actualization (Chen et al., 2022). In this context, the Internet and social media platforms serve as important channels for disseminating anti-natalist perspectives. Individuals with higher levels of generalized trust are more exposed to such heterogeneous viewpoints, which may in turn be associated with weaker fertility intentions (Ning et al., 2022; Adair et al., 2014). These results suggest that different dimensions of social trust are linked to fertility choices through their influence on values and social cognition.

**TABLE 3:** Correlation among fertility intentions and their key predictors (*N* = 5,455).

Variables	Fertility intention	Subjective wellbeing
Institutional trust	0.096***	0.122***
Particularized trust	0.062***	0.089***
Generalized trust	−0.091***	0.055***
Expert-system trust	0.051***	0.062***
Subjective wellbeing	0.052***	

***p < 0.001.

The mediating variable, subjective wellbeing, is positively correlated with fertility intentions (*r* = 0.052, *p* < 0.001). Table 3 also reports correlations between social trust and subjective wellbeing. All four types of social trust are positively and significantly correlated with subjective wellbeing, with institutional trust showing the strongest correlation (*r* = 0.122) and generalized trust the weakest (*r* = 0.055).

### 3.3 Ordinal logistic regression analysis

Using CGSS2023 data, we estimated regression models to examine the association between social trust and fertility intentions (Table 4). A Brant test was conducted to assess the proportional odds assumption. The results showed a chi-square value of 19.61 and a *p*-value of 0.238 (*p* > 0.05), suggesting that the proportional odds assumption is not violated and that the ordinal logistic regression model is appropriate for subsequent analysis.

**TABLE 4:** Ordered logistic regression results of social trust on fertility intentions (*N* = 5,455).

Variables	Model 1		Model 2	
	β	OR	β	OR
Male	0.220*** (0.058)	1.246*** (0.072)	0.221*** (0.058)	1.248*** (0.073)
Age	0.042*** (0.003)	1.043*** (0.002)	0.042*** (0.002)	1.042*** (0.002)
Han nationality	−0.190* (0.094)	0.827* (0.078)	−0.185* (0.095)	0.831* (0.079)
CCP members	0.122 (0.090)	1.130 (0.102)	0.108 (0.091)	1.114 (0.101)
Married	0.383*** (0.065)	1.467*** (0.096)	0.374*** (0.065)	1.453*** (0.095)
Years of education	−0.052*** (0.008)	0.949*** (0.007)	−0.050*** (0.008)	0.951*** (0.007)
Personal income	−0.026*** (0.008)	0.974*** (0.008)	−0.025*** (0.008)	0.975*** (0.008)
Urban	−0.617*** (0.064)	0.538*** (0.034)	−0.572*** (0.064)	0.564*** (0.036)
Employment	0.318*** (0.067)	1.375*** (0.092)	0.295*** (0.067)	1.344*** (0.090)
Health	0.054* (0.027)	1.056* (0.028)	0.049* (0.027)	1.051* (0.028)
Institutional trust			0.199*** (0.050)	1.220*** (0.061)
Particularized trust			0.093* (0.058)	1.097* (0.063)
Generalized trust			−0.066 (0.056)	0.936 (0.052)
Expert-system trust			0.168*** (0.055)	1.184*** (0.065)
Cut1	−1.166 (0.212)		0.247 (0.402)	
Cut2	0.340 (0.209)		1.758 (0.402)	
Cut3	3.398 (0.215)		4.830 (0.407)	
Pseudo R^2^	0.088		0.091	

*p < 0.05, ***p < 0.001.

Model 1 includes only control variables, and the results indicate that most control variables are significantly associated with fertility intentions, except for political affiliation. Specifically, males report stronger fertility intentions than females; Han respondents report weaker fertility intentions than ethnic minorities; married individuals show substantially stronger fertility intentions than unmarried individuals; urban residents exhibit markedly weaker fertility intentions than rural residents; and employed individuals have a stronger fertility intention. Moreover, fertility intentions decline significantly as years of education and personal income increase. Compared to those with lower health levels, individuals with better self-rated health exhibit a stronger fertility intention. The direction differs from the bivariate association reported in the descriptive analysis, suggesting that the relationship between self-rated health and fertility intentions is influenced by other demographic characteristics.

We added the independent variable of social trust in Model 2. The results indicate that institutional trust (β = 0.199, OR = 1.220), particularized trust (β = 0.093, OR = 1.097), and expert-system trust (β = 0.168, OR = 1.184) are all positively and significantly associated with fertility intentions. Specifically, a one-unit increase in institutional trust corresponds to a 22% increase in the odds of reporting a higher level of fertility intentions, holding other variables constant. This may be attributed to the fact that, in recent years, the Chinese government and public discourse have consistently emphasized fertility in relation to long-term demographic and social development goals, and have introduced a series of fertility-supporting policy measures (Fan et al., 2020; Wang et al., 2021). Individuals with higher levels of institutional trust are more likely to endorse and respond to these policy initiatives, thereby exhibiting stronger fertility intentions. Furthermore, higher institutional trust may be associated with reduced perceived uncertainties related to child-rearing, such as education, healthcare, and pension security, making them more willing to incur the costs of childbirth. Although Zheng et al. (2024) reached opposite conclusions, a notable distinction lies in the fact that their research placed greater emphasis on the legacy effects of the one-child policy. From a policy-oriented perspective, these differences may reflect variation in institutional context rather than fundamentally divergent mechanisms. Hypothesis 1 has been supported.

Interpersonal trust yields varying outcomes. Particularized trust shows a statistically significant and positive association, indicating a 9.7% increase in the odds of reporting stronger fertility intentions. In the Chinese social context, expectations from parents and relatives regarding marriage and childbearing are often embedded in everyday social relations. Individuals with higher trust in family members and friends are more inclined to conform to these social expectations, thus increasing their fertility intentions. Moreover, higher particularized trust also provides stronger social support, allowing individuals to rely on material and emotional assistance from family members and friends during child-rearing (Aassve et al., 2012; Thomese and Liefbroer, 2013). This, in turn, potentially reduces the costs and psychological burdens of raising children, thus enhancing the likelihood of childbearing. Rutigliano (2020) also finds that first-time parents anticipate future levels of grandparental support by observing their parents’ characteristics and adjust their decision to enter parenthood accordingly. Hypothesis 2a is therefore supported. In contrast, generalized trust does not exhibit a significant relationship with fertility intentions (*p* > 0.05). This suggests that the association between generalized trust and fertility intentions may be more indirect and not captured in the current specification. Hypothesis 2b is therefore not supported.

Expert-system trust also demonstrates a significant positive association with fertility intentions. Individuals with higher levels of expert-system trust are approximately 18.4% more likely to report stronger fertility intentions. The positive role of expert-system trust primarily relates to individuals’ sense of security in dealing with uncertainties and risks. Such confidence is also associated with stronger expectations regarding child health, safety, and institutional support in child-rearing contexts, which in turn may correspond with stronger fertility intentions. Hypothesis 3 is thus supported.

Finally, additional robustness analyses using ordered probit models yielded substantively similar results (Table 5).

**TABLE 5:** Robustness test (ordered probit models) (*N* = 5,455).

Variables	Model 3
Control variables	Yes
Institutional trust	0.111*** (0.028)
Particularized trust	0.060* (0.033)
Generalized trust	−0.045 (0.032)
Expert-system trust	0.095*** (0.031)

*p < 0.05, ***p < 0.001.

### 3.4 Structural equation model

**FIGURE 3:**
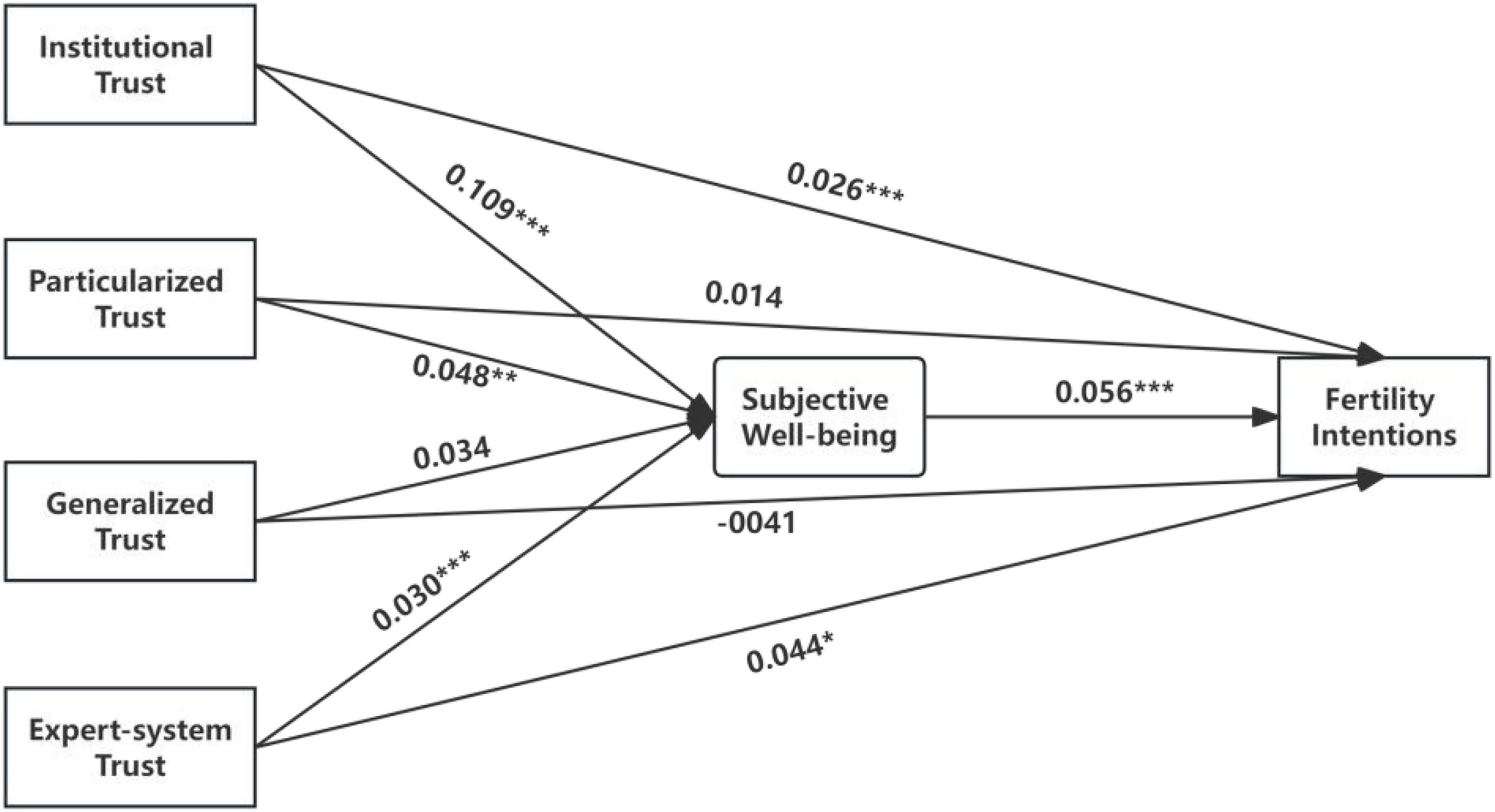
Path diagram of the mediating effect of subjective wellbeing on fertility intentions.

The influence of social trust on fertility intentions is further examined using a structural equation model, with fertility intention as the dependent variable and demographic variables controlled for as specified earlier (Figure 3).

The model results indicate that both institutional trust and expert-system trust are significantly associated with fertility intentions through direct and indirect pathways, with subjective wellbeing as a mediator. High levels of institutional and expert-system trust signify an enhanced sense of security in the social environment and more optimistic expectations about the future, which in turn promote more positive attitudes toward child-rearing. Moreover, these forms of trust are also substantially beneficial to subjective wellbeing, thus providing emotional and psychological support relevant to fertility-related decision-making. These findings underscore the potential importance of institutional environments and expert systems in shaping individuals’ psychological experiences, which ultimately influence fertility preference. Hypotheses 4 and 6 are thus supported.

By contrast, particularized trust does not show a statistically significant direct association with fertility intentions, but it does exhibit a significant indirect association via subjective wellbeing. In other words, particularized trust may not directly motivate stronger fertility intentions; rather, it may be associated with stronger social support networks that may reduce the psychological and material costs of child-rearing, and thereby contribute to subjective wellbeing, subsequently influencing fertility intentions positively. The results of the structural equation model indicate that generalized trust is not significantly correlated with either fertility intentions or subjective wellbeing.

To obtain more robust standard errors and confidence intervals, bootstrap resampling procedures were employed to supplement and validate the mediation analysis. The results indicate that multiple trust dimensions exhibit statistically significant indirect associations with fertility intentions through subjective wellbeing. Subjective wellbeing accounts for approximately 14.1% of the total association between institutional trust and fertility intentions, while it accounts for 21.1% for particularized trust, and 12.3% for expert-system trust. Similarly, the mediating role of subjective wellbeing in the case of generalized trust could not be confirmed.

## 4 Conclusion and discussion

### 4.1 Conclusion

Fertility intentions are widely regarded as an important proximal determinant of reproductive behavior and a micro-level foundation of a country’s overall fertility rate. Based on the nationally representative CGSS2023 data, this study examined the associations between different dimensions of social trust and fertility intentions. Drawing on a multidimensional conceptualization of social trust, we distinguish institutional trust, interpersonal trust, and expert-system trust, and systematically investigate the mediating role of subjective wellbeing.

Several important findings emerged. First, institutional trust and expert-system trust were positively associated with fertility intentions, and these associations were partially mediated by subjective wellbeing. Individuals expressing higher trust in public institutions and professional systems also tended to report stronger fertility intentions, although the estimated associations were modest in magnitude. On the one hand, trust in institutional governance and professional service provision may contribute to individuals’ willingness to consider childbearing by reducing perceived uncertainty and strengthening positive expectations about the future. On the other hand, these two dimensions of social trust were positively associated with individuals’ subjective wellbeing, providing a psychological basis for stronger fertility intentions.

Second, interpersonal trust exhibited heterogeneous associations across its two dimensions. Particularized trust showed no significant direct association with fertility intentions but was indirectly associated with fertility intentions through subjective wellbeing. This result indicates that trust embedded in close social relationships may primarily operate through psychological and emotional support rather than directly influencing reproductive preferences. On the contrary, generalized trust was not significantly associated with fertility intentions in either the direct or indirect models, suggesting that confidence in unfamiliar others may play a comparatively limited role in fertility preference within the contemporary Chinese context.

Finally, subjective wellbeing emerged as an important psychological pathway linking social trust to fertility intentions. The findings indicate that individuals reporting higher levels of social trust also tend to report higher subjective wellbeing, which in turn is associated with stronger fertility intentions. Together, these results highlight the importance of considering both institutional and psychological processes when examining fertility intentions.

### 4.2 Discussion

This study contributes to the growing literature on social trust and fertility in several respects. First, whereas previous studies have generally conceptualized social trust as either a single construct or a broad contextual characteristic, this study adopts a multidimensional perspective by distinguishing institutional trust, interpersonal trust, and expert-system trust. Building on the theoretical perspectives of Luhmann and Giddens, the findings suggest that different forms of trust are associated with fertility intentions through distinct pathways. Although institutional trust and expert-system trust are positively correlated, they are not empirically interchangeable, supporting the theoretical distinction proposed in this study. This finding also responds to recent discussions emphasizing the need to distinguish different objects of trust, particularly in institutional settings where professional systems are closely embedded within state governance.

Second, the findings generally align with previous studies reporting positive associations between institutional trust and fertility intentions (Aassve et al., 2016; Gortfelder et al., 2024), while also illustrating that these relationships may vary across institutional contexts. Unlike earlier studies reporting negative associations between trust in governments and fertility intentions in China (Zheng et al., 2024), the present findings suggest a positive association under the current pronatalist policy environment. One possible explanation is that, following the transition from fertility restriction to fertility promotion, institutional trust increasingly reflects confidence in the government’s capacity to provide family-support policies and public services. Nevertheless, given the cross-sectional nature of the data, these findings should be interpreted as statistical associations rather than evidence of causal effects.

Third, the heterogeneous findings for interpersonal trust provide additional insight into the role of social relationships in fertility decision-making. The indirect association between particularized trust and fertility intentions is consistent with research emphasizing the importance of family support and close social networks in reducing the psychological and practical costs of child-rearing (Philipov et al., 2006). By contrast, the absence of a significant association for generalized trust may reflect the relatively low level of trust toward unfamiliar others in the present sample. However, because the estimated effect sizes are relatively small, this finding should not be interpreted as indicating that generalized trust lacks relevance. Rather, its influence on fertility intentions may operate through mechanisms that are not captured in the present study.

Finally, this study highlights the importance of subjective wellbeing as a psychological mechanism linking social trust and fertility intentions. Consistent with previous research (Aassve et al., 2015; Mencarini et al., 2018), individuals reporting higher levels of subjective wellbeing also tended to report stronger fertility intentions. The mediation analysis further suggests that social trust may be associated with fertility intentions partly through its relationship with subjective wellbeing, emphasizing the importance of integrating institutional, social, and psychological perspectives in explaining reproductive decision-making.

From a practical perspective, the findings suggest that improving fertility intentions requires more than direct economic incentives. Policies that strengthen institutional credibility, improve the quality and accessibility of public services, enhance professional service systems, and reinforce family and community support networks may jointly contribute to creating a more supportive environment for childbearing and child-rearing.

### 4.3 Limitations and future research

Although this study has made some progress, there are still limitations. Future research should be expanded in the following directions: First, this study relies on cross-sectional CGSS data, which precludes strong causal inference. Although the regression and structural equation models identify statistically significant associations among different dimensions of social trust, subjective wellbeing, and fertility intentions, reverse causality and omitted-variable bias cannot be completely ruled out. Future research should employ longitudinal or panel data to verify temporal ordering and causal mechanisms more rigorously. In addition, the measurement of fertility intention in this study reflects ideal fertility preferences rather than short-term reproductive plans. Therefore, future research could further examine whether the associations identified here extend to actual fertility behavior using longitudinal data or samples specifically targeting individuals in reproductive ages.

Second, the relatively close association between institutional trust and expert-system trust may reflect China’s highly institutionalized governance structure, suggesting that future research should further investigate whether these dimensions remain empirically distinguishable across different institutional contexts.

Third, China exhibits substantial regional, socioeconomic, and urban-rural heterogeneity. Future studies could investigate whether the associations identified in this study vary across different geographic regions or population groups.

Fourth, additional psychological and social variables, such as perceived social support, perceived policy effectiveness, and risk perception, may further clarify the mechanisms through which different forms of social trust are associated with fertility intentions.

Fifth, although this study conceptualizes social trust as a multidimensional construct, the measurement of subjective wellbeing remains relatively limited. Future research could employ more comprehensive multidimensional measures to improve construct validity.

Finally, because the present study focuses exclusively on China, the generalizability of the findings to other institutional and cultural contexts remains uncertain. Comparative cross-national research would help determine whether the multidimensional mechanisms identified here are context-specific or more broadly applicable.

## Data Availability

The original contributions presented in the study are included in the article/Supplementary material, further inquiries can be directed to the corresponding author.

## References

[B1] Aassve A. Arpino B. Goisis A. (2012). Grandparenting and mothers’ labour force participation: A comparative analysis using the Generations and Gender Survey. *Demogr. Res.* 27:3. 10.4054/demres.2012.27.3

[B2] Aassve A. Billari F. Pessin L. (2016). Trust and fertility dynamics. *Soc. Forces* 95 663–692. 10.1093/sf/sow080 28003707 PMC5167372

[B3] Aassve A. Mencarini L. Sironi M. (2015). Institutional change, happiness, and fertility. *Eur. Sociol. Rev.* 31 749–765. 10.1093/esr/jcv073

[B4] Adair L. Brase G. Akao K. Jantsch M. (2014). #babyfever: Social and media influences on fertility desires. *Pers. Individ. Dif.* 71 135–139. 10.1016/j.paid.2014.07.026

[B5] Alijanzadeh M. Bahrami N. Jafari E. Noori M. Miri F. Joftyar M. et al.. (2023). Iranian women’s attitude toward childbearing and its’ association with generalized trust, social support, marital satisfaction and governmental childbearing incentives. *Heliyon* 9:e16162. 10.1016/j.heliyon.2023.e16162 37215895 PMC10199260

[B6] Bain K. Hicks N. (1998). Building social capital: The challenge of partnerships. *Int. J. Techn. Cooperation* 4 213–226.

[B7] Berninger I. Weiß B. Wagner M. (2011). On the links between employment, partnership quality, and the desire to have a first child. *Demographic Res.* 24:24. 10.4054/demres.2011.24.24

[B8] Billari F. C. (2009). “The happiness commonality: Fertility decision in low-fertility settings,” in *How Generations and Gender Shape Demographic Change*, ed. UNECE (Geneva: United Nations), 7–38.

[B9] Buber I. Panova R. Dorbritz J. (2013). Fertility intentions of university graduates. *Demográfia* 56 5–34.

[B10] Chen T. Hou P. Wu T. Yang J. (2022). The impacts of the COVID-19 pandemic on fertility intentions of women with childbearing age in China. *Behav. Sci.* 12:335. 10.3390/bs12090335 36135139 PMC9495656

[B11] Comolli C. (2017). The fertility response to the great recession in Europe and the United States: Structural economic conditions and perceived economic uncertainty. *Demogr. Res.* 36:51. 10.4054/demres.2017.36.51

[B12] Fan E. Maitra P. (2010). *Women Rule: Preferences and Fertility in Australian Households*. Department of Economics Discussion Papers No. 5710. Melbourne: Monash University Press, 1–32.

[B13] Fan S. Xiao C. Zhang Y. Li Y. Wang X. Wang L. (2020). How does the two-child policy affect the sex ratio at birth in China? A cross-sectional study. *BMC Public Health* 20:789. 10.1186/s12889-020-08799-y 32460822 PMC7251839

[B14] Fiori F. Rinesi F. Pinnelli A. Prati S. (2013). Economic insecurity and the fertility intentions of Italian women with one child. *Population Res. Policy Rev.* 32 373–413. 10.1007/s11113-013-9266-9

[B15] Fung P. Etienne H. (2023). Confucius, cyberpunk and Mr. Science: Comparing AI ethics principles between China and the EU. *AI Ethics* 3 505–511. 10.1007/s43681-022-00180-6 35756856 PMC9207836

[B16] Gao S. Zhao J. (2022). The influence of perception of social equality and social trust on subjective well-being among rural Chinese people: The moderator role of education. *Front. Psychol.* 12:731982. 10.3389/fpsyg.2021.731982 35046863 PMC8762249

[B17] Ghaheri A. Omani-Samani R. Sepidarkish M. Hosseini M. Maroufizadeh S. (2020). The four-item patient health questionnaire for anxiety and depression: A Validation study in infertile patients. *Int. J. Fertil. Steril.* 14 234–239. 10.22074/ijfs.2020.44412 33098392 PMC7604697

[B18] Giddens A. (1990). *The Consequences of Modernity.* Redwood City, CA: Stanford University Press.

[B19] Giddens A. (2002). *Modernitet och självidentitet – Självet och samhället i den senmoderna epoken. Göteborg: Daidalos.* Swedish.

[B20] Gobbi P. E. A. (2012). model of voluntary childlessness. *J. Population Econ.* 26 963–982. 10.1007/s00148-012-0457-1

[B21] Gortfelder M. Neyer G. Andersson G. (2024). Trust and fertility intentions in high-trust Sweden: An exploratory analysis. *Comp. Populat. Stud.* 49 A1–A14. 10.12765/CPoS-2024-12

[B22] Hanappi D. Ryser V. Bernardi L. Le Goff J. (2017). Changes in employment uncertainty and the fertility intention-realization link: An analysis based on the swiss household panel. *Eur. J. Popul.* 33 381–407. 10.1007/s10680-016-9408-y 28725099 PMC5493711

[B23] Helliwell J. F. Wang S. (2011). Trust and well-being. *Int. J. Wellbeing* 1 42–78. 10.5502/ijw.v1i1.3

[B24] Helliwell J. F. Huang H. Wang S. (2017). “New evidence on trust and well-being,” in *The Oxford Handbook of Social and Political Trust*, ed. Uslaner E. M. (Oxford: Oxford University Press). 10.1093/oxfordhb/9780190274801.013.9

[B25] Helliwell J. F. Layard R. Sachs J. D. (2012). *World Happiness Report.* New York, NY: The Earth Institute, Columbia University. Available online at: http://eprints.lse.ac.uk/47487/

[B26] Helliwell J. Putnam R. (2004). The social context of well-being. *Philos. Trans. R. Soc. Lond. B Biol. Sci.* 359 1435–1446. 10.1098/rstb.2004.1522 15347534 PMC1693420

[B27] Hensvik L. Nilsson P. (2010). *Businesses, Buddies and Babies: Social Ties and Fertility at Work*. Working Paper Series. 9. Uppsala: IFAU-Institute for Evaluation of Labour Market and Education Policy.

[B28] Hin S. Gauthier A. Goldstein J. Bühler C. (2011). Fertility preferences: What measuring second choices teaches us. *Vienna Yearbook Population Res.* 9 131–156. 10.1553/populationyearbook2011s131

[B29] Hobcraft J. Kiernan K. (1995). “Becoming a parent in Europe,” in *European Population Conference* (Plenary sessions), 27–65.

[B30] Jiang Q. Li Y. Sanchez-Barricarte J. (2016). Fertility intention, son preference, and second childbirth: Survey findings from shaanxi province of China. *Soc. Indic. Res.* 125 935–953. 10.1007/s11205-015-0875-z 28769144 PMC5536174

[B31] Kaasa A. Andriani L. (2022). Determinants of institutional trust: The role of cultural context. *J. Institutional Econ.* 18 45–65. 10.1017/s1744137421000199

[B32] Knack S. (2002). Social capital and the quality of government: Evidence from the states. *Am. J. Polit. Sci.* 46:772. 10.2307/3088433

[B33] Kohler H. Billari F. Ortega J. (2002). The emergence of lowest-low fertility in Europe during the 1990s. *Population Dev. Rev.* 28 641–680. 10.1111/j.1728-4457.2002.00641.x

[B34] Kotte M. Ludwig V. (2011). Intergenerational transmission of fertility intentions and behaviour in Germany: The role of contagion. *Vienna Yearbook Population Res.* 9 207–226.

[B35] Kuhnt A. Trappe H. (2016). Channels of social influence on the realization of short-term fertility intentions in Germany. *Adv. Life Course Res.* 27 16–29. 10.1016/j.alcr.2015.10.002

[B36] Kuroki M. (2011). Does social trust increase individual happiness in Japan?*. *Jpn. Econ. Rev.* 62 444–459. 10.1111/j.1468-5876.2011.00533.x

[B37] Kwag H. Baek S. Kang M. (2024). Impact of social trust on young adults’ childbearing intention: Why does South Korea show ultra-low fertility? *Int. J. Urban Sci.* 28 585–610. 10.1080/12265934.2024.2320905

[B38] Lau B. Huo R. Wang K. Shi L. Li R. Mu S. et al.. (2018). Intention of having a second child among infertile and fertile women attending outpatient gynecology clinics in three major cities in China: A cross-sectional study. *Hum. Reprod. Open* 2018:hoy014. 10.1093/hropen/hoy014 30895255 PMC6276692

[B39] Li N. He M. (2022). Social security satisfaction and people’s subjective wellbeing in China: The serial mediation effect of social fairness and social trust. *Front. Psychol.* 13:855530. 10.3389/fpsyg.2022.855530 35444600 PMC9014885

[B40] Li X. (2023). Analysis of influencing factors of fertility intention based on logistic model. *Acad. J. Manag. Soc. Sci.* 2 72–76. 10.54097/ajmss.v2i2.7537

[B41] Lu H. Tong P. Zhu R. (2020). Longitudinal Evidence on social trust and happiness in China: Causal effects and mechanisms. *J. Happ. Stud.* 21 1841–1858. 10.1007/s10902-019-00159-x

[B42] Luhmann N. (1968). *Vertrauen: Ein Mechanismus der Reduktion sozialer Komplexität.* Stuttgart: F. Enke Verlag.

[B43] Mencarini L. Vignoli D. Zeydanli T. Kim J. (2018). Life satisfaction favors reproduction. The universal positive effect of life satisfaction on childbearing in contemporary low fertility countries. *PLoS One* 13:e0206202. 10.1371/journal.pone.0206202 30517115 PMC6281189

[B44] Ning C. Wu J. Ye Y. Yang N. Pei H. Gao H. (2022). How media use influences the fertility intentions among chinese women of reproductive age: A perspective of social trust. *Front. Public Health* 10:882009. 10.3389/fpubh.2022.882009 35619808 PMC9127136

[B45] Perelli-Harris B. (2006). The influence of informal work and subjective well-being on childbearing in post-Soviet Russia. *Population Dev. Rev.* 32 729–753. 10.1111/j.1728-4457.2006.00148.x

[B46] Philipov D. Spéder Z. Billari F. (2006). Soon, later, or ever? The impact of anomie and social capital on fertility intentions in Bulgaria (2002) and Hungary (2001). *Popul. Stud.* 60 289–308. 10.1080/00324720600896080 17060055

[B47] Rosina A. Testa M. (2009). Couples’ first child intentions and disagreement: An analysis of the Italian case. *Eur. J. Populat.* 25 487–502. 10.1007/s10680-009-9188-8

[B48] Rutigliano R. (2020). Counting on potential grandparents? Adult children’s entry into parenthood across European countries. *Demography* 57 1393–1414. 10.1007/s13524-020-00890-8 32519304 PMC7441078

[B49] Schoen R. Astone N. Kim Y. Nathanson C. Fields J. (1999). Do fertility intentions affect fertility behavior? *J. Marriage Fam.* 61:790. 10.2307/353578

[B50] Suriyanrattakorn S. Chang C. (2021). Valuation of trust in government: The wellbeing valuation approach. *Sustainability* 13:11000. 10.3390/su131911000

[B51] Tan N. Anwar S. (2025). The moderating role of happiness in the relationship between fertility desire and behavior in China. *Humanities Soc. Sci. Commun.* 12:1558. 10.1057/s41599-025-05871-z

[B52] Testa M. R. Stephany F. (2017). The educational gradient of fertility intentions: A meta-analysis of European studies. *Vienna Yearbook Population Res.* 15 293–330. 10.1553/populationyearbook2017s293

[B53] Testa M. Rampazzo F. (2021). Intentions and childbearing. *Institut für Demographie - VID.* 1 1–26. 10.1553/0x003cd00a

[B54] Thomese F. Liefbroer A. (2013). Child care and child births: The role of grandparents in the Netherlands. *J. Marriage Fam.* 75 403–421. 10.1111/jomf.12005

[B55] Tokuda Y. Fujii S. Inoguchi T. (2010). Individual and Country-level effects of social trust on happiness: The Asia barometer survey. *J. Appl. Soc. Psychol.* 40 2574–2593. 10.1111/j.1559-1816.2010.00671.x

[B56] Valgarğsson V. Jennings W. Stoker G. Bunting H. Devine D. McKay L. et al.. (2025). Crisis of political trust? global trends in institutional trust from 1958 to 2019. *Br. J. Polit. Sci.* 55:e15. 10.1017/s0007123424000498

[B57] Wang D. Li H. Liu P. (2025). The impact of neighborhood environment and social interaction on the health of Chinese residents: Empirical analysis from CGSS 2021. *Front. Public Health* 13:1547499. 10.3389/fpubh.2025.1547499 40401069 PMC12093411

[B58] Wang Y. Kong F. Qiao J. (2021). Gender equity, caregiving, and the 1-2-3-child policy in China - Authors’ reply. *Lancet* 398 953–954. 10.1016/S0140-6736(21)01749-9 34509225

[B59] Waren W. Pals H. (2013). Comparing characteristics of voluntarily childless men and women. *J. Population Res.* 30 151–170. 10.1007/s12546-012-9103-8

[B60] Yamamura E. Antonio R. A. (2011). *Trust and fertility: Evidence from OECD countries. Munich Personal RePEc Archive.* Available online at: https://mpra.ub.uni-muenchen.de/29978 (accessed July 23, 2026).

[B61] Yang Q. Tang W. (2010). Exploring the sources of institutional trust in China: Culture, mobilization, or performance? *Asian Polit. Policy* 2 415–436. 10.1111/j.1943-0787.2010.01201.x

[B62] Yang Y. He R. Zhang N. Li L. (2023). Second-child fertility intentions among urban women in China: A systematic review and meta-analysis. *Int. J. Environ. Res. Public Health* 20:3744. 10.3390/ijerph20043744 36834437 PMC9962327

[B63] Yeh M. (2022). Intergenerational contract in ageing democracies: Sustainable welfare systems and the interests of future generations. *Med. Health Care Philos.* 25 531–539. 10.1007/s11019-022-10098-9 35697971

[B64] Yu W. Kuo J. (2017). Another work-family interface: Work characteristics and family intentions in Japan. *Demographic Res.* 36:13. 10.4054/demres.2017.36.13

[B65] Zhang T. Cai X. Shi X. Zhu W. Shan S. (2023). The effect of family fertility support policies on fertility, their contribution, and policy pathways to fertility improvement in OECD countries. *Int. J. Environ. Res. Public Health* 20:4790. 10.3390/ijerph20064790 36981698 PMC10049131

[B66] Zheng J. Wang X. Xie S. Wang H. Shen J. Zhang T. (2024). The mediating role of trust in government in intergenerational transmission of fertility intentions. *Front. Public Health* 12:1338122. 10.3389/fpubh.2024.1338122 38496397 PMC10941980

[B67] Zhou M. Guo W. (2020). Fertility intentions of having a second child among the floating population in China: Effects of socioeconomic factors and home ownership. *Popul. Space Place* 26:2289. 10.1002/psp.2289

[B68] Zhou X. Chen S. Chen L. Li L. (2021). Social class identity, public service satisfaction, and happiness of residents: The mediating role of social trust. *Front. Psychol.* 12:659657. 10.3389/fpsyg.2021.659657 34140917 PMC8203815

